# MARTINI bead form factors for the analysis of time-resolved X-ray scattering of proteins

**DOI:** 10.1107/S1600576714009959

**Published:** 2014-06-14

**Authors:** Stephan Niebling, Alexander Björling, Sebastian Westenhoff

**Affiliations:** a Department of Chemistry and Molecular Biology, University of Gothenburg, Box 462, SE-40530 Gothenburg, Sweden

**Keywords:** X-ray solution scattering, proteins, coarse-graining, MARTINI, structural dynamics, small-angle X-ray scattering (SAXS), wide-angle X-ray scattering (WAXS), protein structure refinement

## Abstract

Time-resolved small- and wide-angle X-ray scattering (SAXS and WAXS) methods probe the structural dynamics of proteins in solution. Although technologically advanced, these methods are in many cases limited by data interpretation. The calculation of X-ray scattering profiles is computationally demanding and poses a bottleneck for all SAXS/WAXS-assisted structural refinement and, in particular, for the analysis of time-resolved data. A way of speeding up these calculations is to represent biomolecules as collections of coarse-grained scatterers. Here, such coarse-graining schemes are presented and discussed and their accuracies examined. It is demonstrated that scattering factors coincident with the popular MARTINI coarse-graining scheme produce reliable difference scattering in the range 0 < *q* < 0.75 Å^−1^. The findings are promising for future attempts at X-ray scattering data analysis, and may help to bridge the gap between time-resolved experiments and their interpretation.

## Abstract 2

Form factors for X-ray scattering calculations from coarse-grained MARTINI protein models are derived. The reliability at different levels of coarse-graining is evaluated and weighed against the gain in computational speed of the coarser models.

## 1. Introduction   

X-ray solution scattering is a popular technique for gathering structural information on biomolecules in solution (Petoukhov & Svergun, 2007 ▶; Koch *et al.*, 2003 ▶; Svergun & Koch, 2003 ▶; Makowski, 2010 ▶; Ihee *et al.*, 2010 ▶; Westenhoff *et al.*, 2010 ▶; Andersson *et al.*, 2009 ▶; Cho *et al.*, 2010 ▶; Malmerberg *et al.*, 2011 ▶; Kim, Muniyappan *et al.*, 2012 ▶; Kim, Lee *et al.*, 2012 ▶; Ibrahimkutty *et al.*, 2011 ▶; Spilotros *et al.*, 2012 ▶; Takala *et al.*, 2014 ▶). The angular intensity distribution of scattered X-rays is recorded and advanced computational algorithms are available to determine three-dimensional structures from the scattering patterns (Konarev *et al.*, 2006 ▶; Petoukhov *et al.*, 2012 ▶; Liu *et al.*, 2012 ▶). X-ray scattering at small angles (SAXS) provides information on molecular envelopes. At wider angles (WAXS), higher-resolution information is encoded, but low scattering strength and a lack of uniqueness when assigning structural features to the data hinders its practical application.

Time-resolved X-ray solution scattering is an emerging technique for observing structural changes of proteins (Ihee *et al.*, 2010 ▶; Westenhoff *et al.*, 2010 ▶; Andersson *et al.*, 2009 ▶; Makowski, 2010 ▶; Cho *et al.*, 2010 ▶; Malmerberg *et al.*, 2011 ▶; Kim, Muniyappan*et al.*, 2012 ▶; Kim, Lee *et al.*, 2012 ▶; Ibrahimkutty *et al.*, 2011 ▶; Spilotros *et al.*, 2012 ▶; Takala *et al.*, 2014 ▶). X-ray scattering is recorded as a function of reaction time and referenced to the scattering patterns of the reactants. The difference technique makes it possible to access higher spatial resolution by detecting WAXS, since all background signals are very precisely canceled. At modern synchrotron facilities the time resolution is limited to approximately 100 ps, but free-electron laser sources increase the resolution to <100 fs. This opens up the way for studies of elementary structural changes in proteins on the time scale of atomic motions.

Today the bottleneck in protein solution X-ray scattering lies in interpreting the experimental data. One is forced to model it in an iterative fashion, and to calculate scattering pat­terns of a large number of trial structures. Since the total scat-tering is a result of pairwise interference between all the atoms in a protein, each such calculation is time consuming and refinement quickly becomes too computationally demanding.

For a realistic representation of scattering from a molecule in solution, the contributions to the form factor from the electron density of the molecule, the electron density of the displaced solvent and any excess electron density of the solvation shell have to be evaluated. The first term is often computed from the atomic coordinates of the molecule. To represent the solvent displaced by the solute, it is common practice to use modified atomic scattering factors (Fraser *et al.*, 1978 ▶). Whereas the use of this approximation is justified at small angles, systematic deviations are introduced for higher angles (Bardhan *et al.*, 2009 ▶). The excess electron density in the solvation shell is often modeled as a homogeneous border layer, as implemented in the popular program *CRYSOL* (Svergun *et al.*, 1995 ▶). However this strategy is problematic as two parameters describing the solvation shell are introduced and adjusted *ad hoc*. Recent developments include explicit solvent treatment (Grishaev *et al.*, 2010 ▶; Park *et al.*, 2009 ▶) or a more realistic representation of the solute–solvent boundary (Bardhan *et al.*, 2009 ▶; Virtanen *et al.*, 2011 ▶). By using these more sophisticated methods, the reliability *q* range can be extended to higher values. However these methods are computationally demanding and it is hard to prove their accuracy experimentally. We show explicitly below that the need for accurate computation of the solvent layer is relaxed when analyzing difference X-ray scattering.

Given that solution scattering signals of proteins do not encode enough information to reveal atomic level details, a coarse-grained representation should often be suitable for interpreting SAXS and WAXS data. Such representations greatly decrease the computational cost of predicting X-ray scattering curves (Yang *et al.*, 2009 ▶; Stovgaard *et al.*, 2010 ▶; Zheng & Tekpinar, 2011 ▶; Daily *et al.*, 2012 ▶), rendering ambitious iterative refinement schemes realistic for relatively large protein systems. In recent years, the MARTINI model, based on coarse-grained representations of biomolecules, has become popular for simulating the dynamics of various biological systems (Marrink *et al.*, 2007 ▶; Monticelli *et al.*, 2008 ▶; López *et al.*, 2009 ▶; Yesylevskyy *et al.*, 2010 ▶; de Jong *et al.*, 2013 ▶; Marrink & Tieleman, 2013 ▶). It drastically reduces the computational cost of molecular dynamics simulations, allowing simulation on longer time scales and of larger systems with modest computational resources. The force field is designed to reproduce thermodynamic data and has been successfully applied to several simulation problems, for example, protein–lipid interactions (van den Bogaart *et al.*, 2011 ▶; Schäfer *et al.*, 2011 ▶; Louhivuori *et al.*, 2010 ▶).

In this study, we describe and compare methods for coarse-grained X-ray scattering calculations, especially aiming at the analysis of time-resolved difference scattering. We show how difference X-ray scattering profiles can be calculated efficiently from MARTINI coarse-grained representations of proteins and we assess the reliability limits for these calculations. We find that coarse-grained scattering calculations are reliable in a larger *q* range for difference scattering compared with absolute scattering. We conclude that this method opens up a way for structural refinement routines of large proteins, especially in combination with time-resolved SAXS/WAXS experiments.

## 2. Theory and methods   

### 2.1. X-ray scattering from coarse-grained structures   

The scattering amplitude from a collection of point-like atomic scatterers (Warren, 1990 ▶) is described by 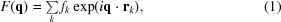
where **q** is the scattering vector and 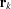 and 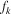 are the position and the scattering factor of atom *k*, respectively. For randomly oriented molecules in solution, the scattered intensity is obtained by multiplying this sum by its complex conjugate,
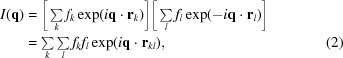
and then taking the spherical average with 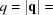 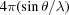, where λ is the wavelength of the radiation, 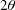 is the scattering angle and 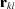 is the vector from scatterer *l* to scatterer *k*. Equation (2) becomes
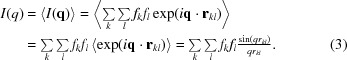
The last result is known as the Debye equation and can be used to predict the vacuum scattering of a biomolecule.

A complication is that solution scattering patterns contain a large undesired solvent signal. This signal can be removed by subtracting a buffer background, but at the cost of including a negative term for the displaced solvent that must be accounted for in predicted data. It can be introduced in an approximate way, at the level of the atomic scattering factors, so that the unmodified Debye equation [equation (3)] can still be used. Such corrected atomic scattering factors 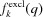 are derived from 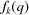 by subtracting a Gaussian sphere representing the scattering amplitude of the displaced solvent (Fraser *et al.*, 1978 ▶): 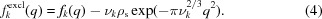
Here 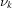 is the tabulated (Fraser *et al.*, 1978 ▶) volume for each atom and 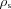 is the mean electron density of the bulk solvent. In the remainder of this paper, all scattering factors contain this displaced-solvent term unless stated otherwise.

For biomolecules, the computational cost for evaluating equation (3) can be quite high. This is especially important for iterative structural refinement procedures where many test structures have to be evaluated. One strategy for decreasing computational cost is to use a coarse-grained representation of the structure, where each coarse bead represents a group of atoms. It is convenient if each bead is described by a scattering factor *F*(*q*), so that the scattering intensity is given by a coarse-grained Debye equation, where the indices (*m*, *n*) denote coarse beads and 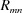 the distance between them: 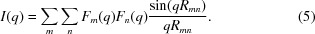
Finding these 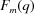 is not trivial and we review some possibilities before describing the approach taken in this study.

#### 2.1.1. The bead position approximation   

The simplest way to express the overall scattering in terms of coarse-grained scattering factors is to consider each atom to be located at the center of its bead. All atom–atom distances are then approximated by the corresponding bead–bead distances. Where (*k*, *l*) are atomic indices and (*m*, *n*) denote coarse beads, the Debye equation can be written as follows:
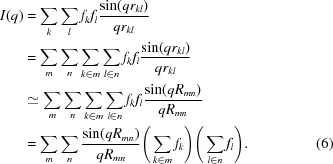
We then have 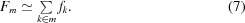
In this approximation, the internal structure of the beads is completely ignored. We note that equation (7) holds exactly for *q* = 0.

#### 2.1.2. The spherical ‘glob’ approximation   

Another option is to take, for each bead, the spherical average of the amplitude [equation (1)] before taking intensities. This is equivalent to smearing each atom out on a sphere of radius 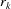 around the center of the bead (Harker, 1953 ▶):
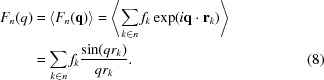
Here, the distance of each atom to the center of the beads is considered, but the angular arrangement of the atoms is ignored.

#### 2.1.3. The self-consistent set approximation   

The most general approach described in this paper is to find, for a given set of proteins, a self-consistent set of *F*(*q*) values that reproduces all pairwise bead–bead scattering intensity terms of the corresponding atomistic structures as well as possible. Considering two beads with scattering factors *F*_A_ and *F*_B_, the total scattering intensity of the pair *I*_AB_ is given by equation (3). These quantities are related by applying equation (5) to the pair of beads: 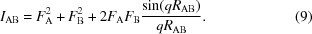
If *F*_B_ is held constant in the comparison, *F*_A_ is obtained by solving this quadratic equation, choosing the correct root by comparing to equation (7), which actually holds for *q* = 0. A self-consistent set of coarse-grained scattering factors can be found from the following scheme.

(*a*) Generate starting guesses for the bead form factors, for example, by using equations (7) or (8).

(*b*) Pick a random bead in the structure and call it A.

(*c*) Go through all other beads in the structure, letting each act as B, and calculate *F*_A_ for each case according to equation (9).

(*d*) Take the average of all these *F*_A_, and assign it to bead A.

(*e*) Repeat (*b*)–(*d*) until the set form factors have converged.

#### 2.1.4. The single-bead approximation   

**Figure 1:**
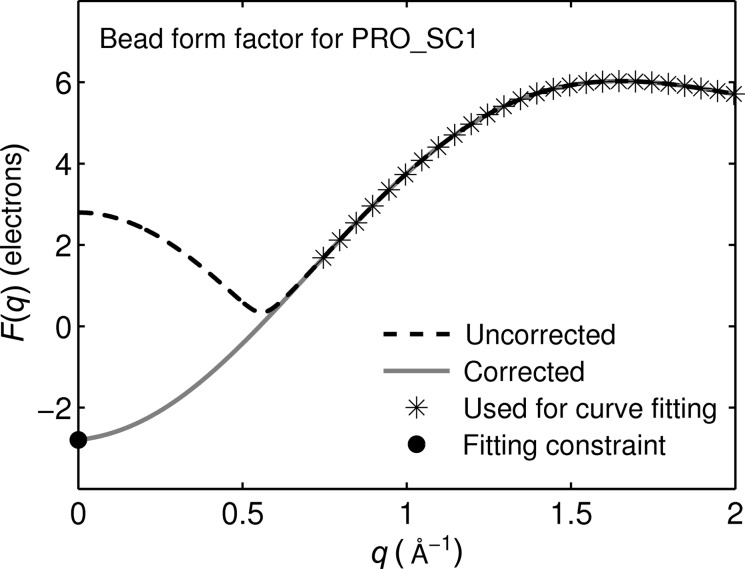
Example of bead scattering factor correction (proline, side chain 1): the dashed curve is calculated according to equation (10). The value for *q* = 0 does not satisfy equation (11) (*cf*. Table 1 ▶). Therefore only the points after the inflection point (following the minimum) were used for the sixth-order polynomial curve fitting (stars). The value at *q* = 0 (from Table 1 ▶) is used as an equality constraint (filled circle). This yields the corrected bead scattering factor (solid line).

Although conceptually simple, the last approach is cumbersome, especially for large libraries of proteins. Yang *et al.* (2009 ▶) have presented a simpler approach, where form factors are chosen such that they reproduce the scattering intensities of isolated beads. In this approach, numerically correct coarse-grained form factors for entire amino acid residues can be obtained simply by taking the square root of the scattering intensity from a group of atoms: 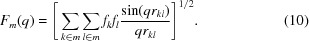
We note that this equation can only produce positive form factors, which is not correct in general when the negative term for the displaced solvent is included. For *q* = 0, the value of the form factor of the bead must equal the sum of the atomic scattering factors: 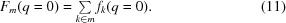



With water as a solvent, *f*_*k*_(*q* = 0) is negative for the hydrogen atom when the displaced-solvent contribution is included. Thus, negative values of *F*(*q*) can occur for beads containing hydrogen atoms [*f*(*q* = 0) = −0.72 electron units (e.u.)]. Therefore, form factors obtained using equation (10) that do not satisfy equation (11) must be corrected. In practice, this is the case for side-chain beads which contain only hydrogen and carbon atoms. When the beads consist of entire amino acids the *f*(*q* = 0) values are generally positive (Yang *et al.*, 2009 ▶). To correct the scattering factors that do not satisfy equation (11), we use a common feature of all these scattering factors, which is the appearance of a minimum with two associated inflection points (Fig. 1 ▶). The data with *q* larger than the high-*q* inflection point are used for a sixth-order polynomial fit, constrained at *q* = 0 to satisfy equation (11). This polynomial is then accepted as the actual form factor of the coarse bead (Fig. 1 ▶).

**Figure 2:**
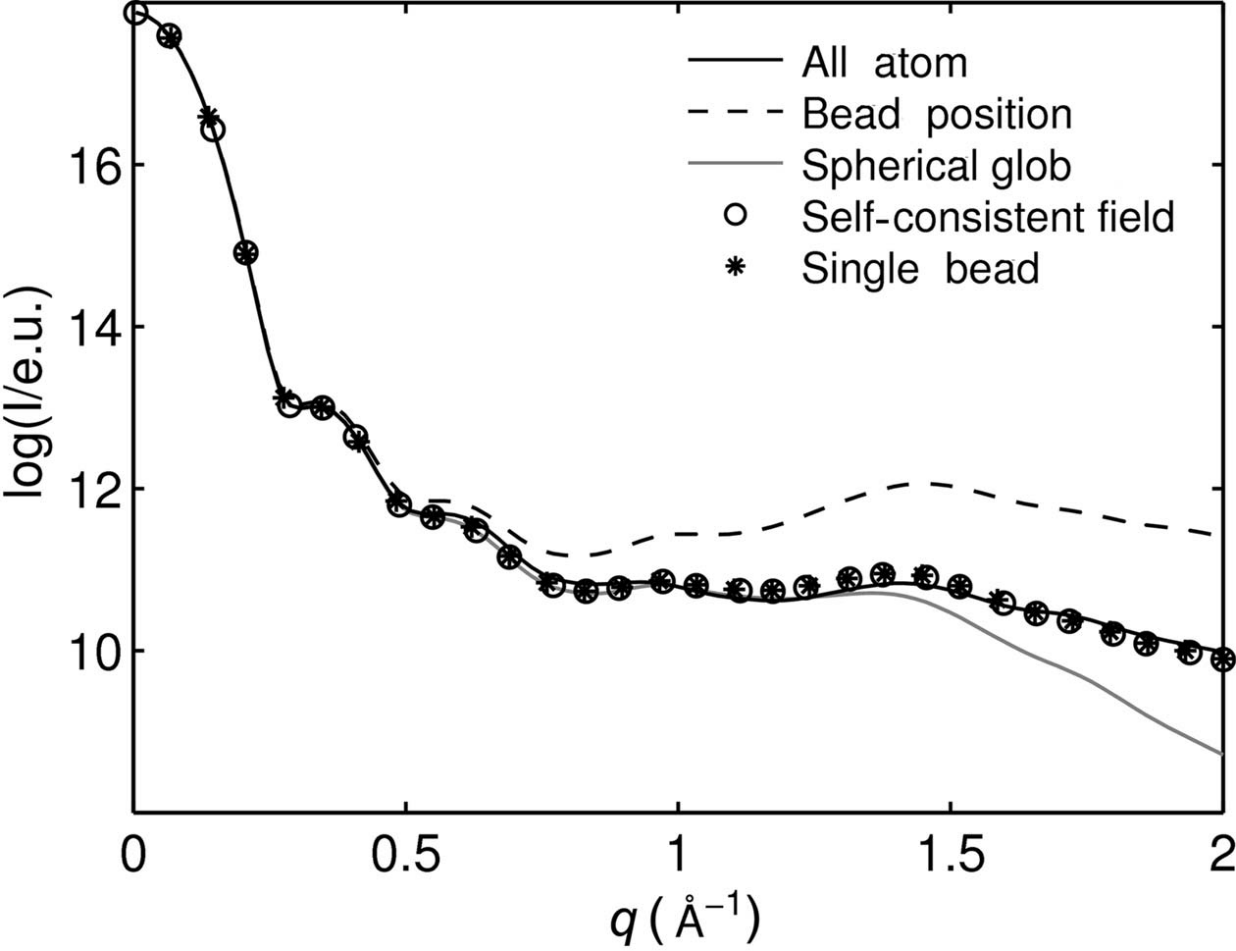
Comparison of the four ways of determining coarse-grained form factors. All calculations were performed without the displaced-solvent model for hen egg-white lysozyme (PDB code 6lyz).

To illustrate the differences between the above four approaches, the coarse-grained X-ray scattering of hen egg-white lysozyme was calculated from a MARTINI (the MARTINI coarse-grained representation of proteins will be described later in §2.2) coarse-grained structure and compared with the all-atom calculation (Fig. 2 ▶). To enable a direct comparison of the form factor calculation methods, the scattering was calculated without the displaced-solvent model. For *q* < 0.25 Å^−1^, all approaches are in good agreement with the all-atom calculation. This is reasonable because long inter-bead distances are probed in this *q* range. Regarding the high-*q* region, the bead-position approximation shows significant deviations for *q* > 0.4 Å^−1^. The spherical glob approximation reproduces the all-atom calculation well for *q* < 1.2 Å^−1^ but deviates for larger *q* values. The self-consistent set approximation and the single-bead approximation yield almost identical results and agree relatively well with the all-atom calculations even for high *q* values.

This degree of agreement corresponds to the order with which the internal structure of the bead is accounted for: the bead-position approximation neglects the internal structure, the spherical glob approximation smears the atoms out on spheres around its center, and the single-bead approximation as well as the self-consistent set approximation include the internal bead structure most accurately. The latter two approximations agree remarkably well with the all-atom calculation. The single-bead approximation is computationally less expensive than the self-consistent set approximation. We therefore chose to use the former for calculating coarse-grained scattering factors in the remainder of this study.

### 2.2. Application to the MARTINI model   

We now turn our attention to applying the single-bead approximation to the MARTINI model (Marrink *et al.*, 2007 ▶; de Jong *et al.*, 2013 ▶; Marrink & Tieleman, 2013 ▶) as an efficient way to calculate X-ray scattering from coarse-grained structures. In the MARTINI model, four non-hydrogen atoms and their associated hydrogen atoms are mapped, on average, onto one bead, with each amino acid residue composed of a backbone bead and up to four side-chain beads (Monticelli *et al.*, 2008 ▶). The beads are grouped by their polarity and hydrogen bonding ability, yielding a total of 20 different bead types whose interactions are specified by the MARTINI force field.

For X-ray scattering calculations the geometrical similarity and the molecular formula (number of electrons) of the beads is of main importance, not the polarity or hydrogen bonding ability. We therefore derive X-ray form factors for each MARTINI bead as it appears in every amino acid residue type. This yields the 49 different scattering types listed in Table 1 ▶. The original mapping of atoms into beads according to the MARTINI model is retained for the X-ray scattering calculations. This means that MARTINI coarse-grained structures can be used directly as an input for these calculations.

The average form factors for the MARTINI beads following the single-bead approximation were acquired from a rationally selected library of protein structures that covers a wide range of protein folds and different secondary structure contents (Oberg *et al.*, 2003 ▶). We excluded seven proteins with missing non-hydrogen atoms. This resulted in a library of 43 proteins shown in Table 2 ▶. Missing hydrogen atoms were added with the pdb2gmx tool which is part of the *GROMACS* suite (Hess *et al.*, 2008 ▶). To investigate the effect of the bead size on accuracy, an additional coarse-grained mapping with one amino acid per bead was used (Yang *et al.*, 2009 ▶; Zheng & Tekpinar, 2011 ▶). Both the amino acid and the MARTINI beads were positioned at the centers of mass of the respective atom groups.

To keep these calculations simple and universally applicable a few structural details were ignored. First of all, the N- and C-termini were not differentiated for the coarse-grained calculation. The respective amino acids were included in the calculation of the average bead scattering factors, and thus the larger number of electrons for the C-terminus is reflected by this averaging. Charged atoms were also ignored, both to limit the number of bead types and since information on the charge is not always available.

## 3. Results   

### 3.1. The library average of bead form factors   

A central aim of this work is to estimate the reliability of coarse-grained X-ray scattering calculations with respect to the size of the beads. Two coarse-grained mapping schemes were used: the amino acid mapping as used by Yang *et al.* (2009 ▶) and our MARTINI-bead approach. A first comparison can be made at the stage of form factor averaging. The smaller the variation between the individual form factors being averaged, the greater the reliability of the coarse-grained scattering calculation. This is especially important for small proteins or proteins of unusual structure or amino acid content.

**Figure 3:**
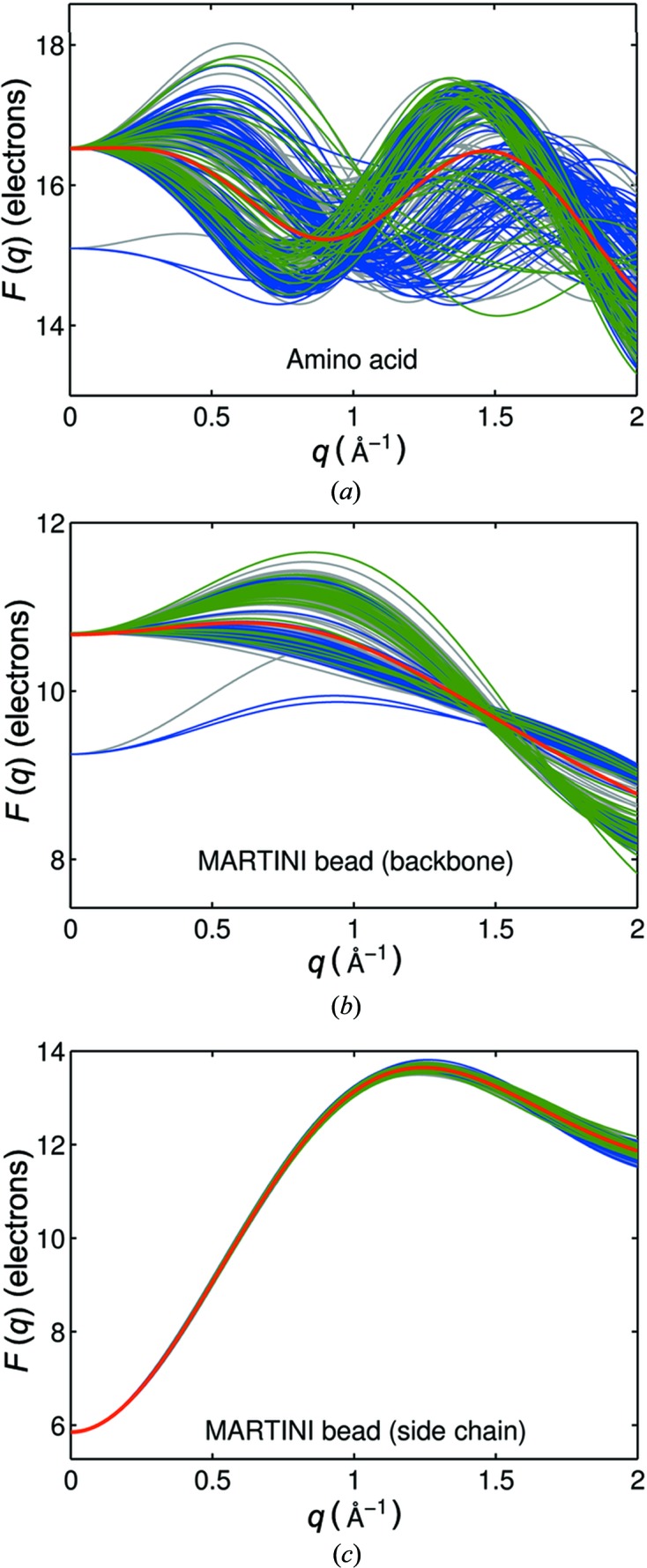
Form factors calculated with displaced solvent for all methionines in the library with amino acid (*a*) and MARTINI bead (*b*), (*c*) coarse-graining. Bead form factors are colored according to the prevalent protein secondary structure motif (blue: α-helical proteins; green: β-sheet proteins; gray: unique assignment not possible). The red curves represent the average over all individual form factors.

The bead scattering factors for all methionines in the library are shown in Fig. 3 ▶. The data were computed with equation (10). Deviations for the amino acid bead and the backbone MARTINI bead at *q* = 0 are the result of the inclusion of N-terminal amino acids, which contain two additional hydrogen atoms. Since the hydrogen scattering with the displaced-solvent correction is negative for low *q*, the corresponding bead scattering factors for low *q* are below the majority of the curves.

The calculated form factors for the amino acid beads show a large variation (Fig. 3 ▶ *a*), whereas the form factors of the finer MARTINI beads are more homogeneous (Figs. 3 ▶ *b* and 3*c*). When the two MARTINI beads (backbone and side chain) are compared, the backbone bead scattering factors are more heterogeneous, whereas the side-chain scattering factors are remarkably well represented by the mean value.

To identify the structural origin of the variation in bead scattering factors, we clustered the protein library based on their prevailing secondary structure. According to the classification of Oberg *et al.* (2003 ▶), the scattering factors of the proteins that show a high percentage of α-helices and β-sheets are shown in different colors (Fig. 3 ▶). The MARTINI backbone bead scattering factors clearly cluster into two groups according to secondary structures. In contrast, the amino acid beads do not show such a clear picture.

### 3.2. Calculation of difference scattering from coarse-grained protein structures   

**Figure 4:**
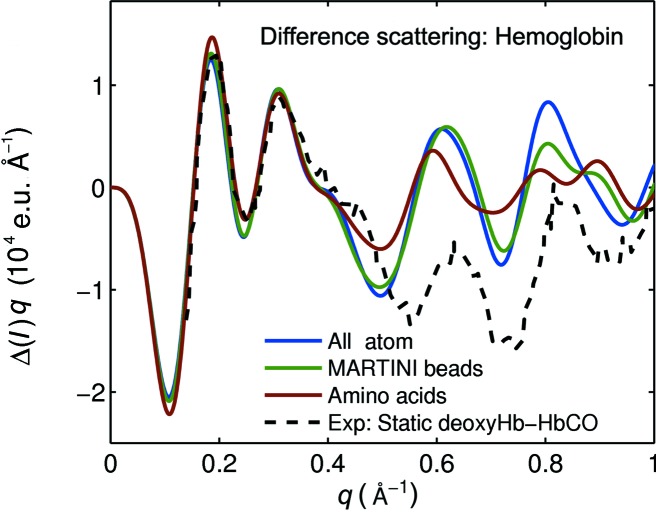
Calculated solution difference scattering between human deoxy and carbonmonoxy hemoglobin crystal models (2hhb–1bbb), compared with experimental solution data from Cammarata *et al.* (2008 ▶). The cofactors were not taken into account in the calculation. The experimental curve has been scaled to the calculated data at *q* = 0.18 Å^−1^.

The analysis of time-resolved WAXS experiments often requires repeated evaluation of difference scattering from many different trial structures and thus depends on fast but reliable scattering calculations over a *q* range up to approximately 1 Å^−1^. In order to estimate the accuracy of difference scattering calculations from coarse-grained structures, the predicted difference scattering between the crystal structures of human deoxy hemoglobin (PDB code 2hhb; Fermi *et al.*, 1984 ▶) compared with human carbonmonoxy hemoglobin (PDB code 1bbb; Silva *et al.*, 1992 ▶), as calculated with all-atom and coarse-grained methods, are shown in Fig. 4 ▶. This model system has already been used for time-dependent X-ray scattering studies and high-quality data are available (Cammarata *et al.*, 2008 ▶). The amino-acid-based result deviates considerably from the all-atom calculation for *q* > 0.4 Å^−1^, but the MARTINI coarse-grained calculation is accurate for *q* < 0.75 Å^−1^. This finding is reasonable considering that the level of structural detail is highest in the all-atom representation, reduced in the MARTINI coarse-graining scheme and lowest in the amino acid approach. The computation times for the three curves in general scale approximately as 50 (all atom):1 (MARTINI):0.2 (amino acid approach).

Comparing the model calculations with the experimental difference scattering from Cammarata *et al.* (2008 ▶) shows that the agreement between the structural model and the experiment is excellent for *q* < 0.4 Å^−1^, but that the model fails for higher *q* values. This is most likely because the crystal structure does not represent the solution structure of hemoglobin very well (Cammarata *et al.*, 2008 ▶). It is obvious that the MARTINI coarse-grained calculations could be used for any refinement algorithm to improve the agreement, but that the amino acid approach would not contain enough structural detail to achieve this. Conversely, such refinement schemes are very likely to benefit from the reduced computational cost of the MARTINI representation relative to the atomistic scattering model.

**Figure 5:**
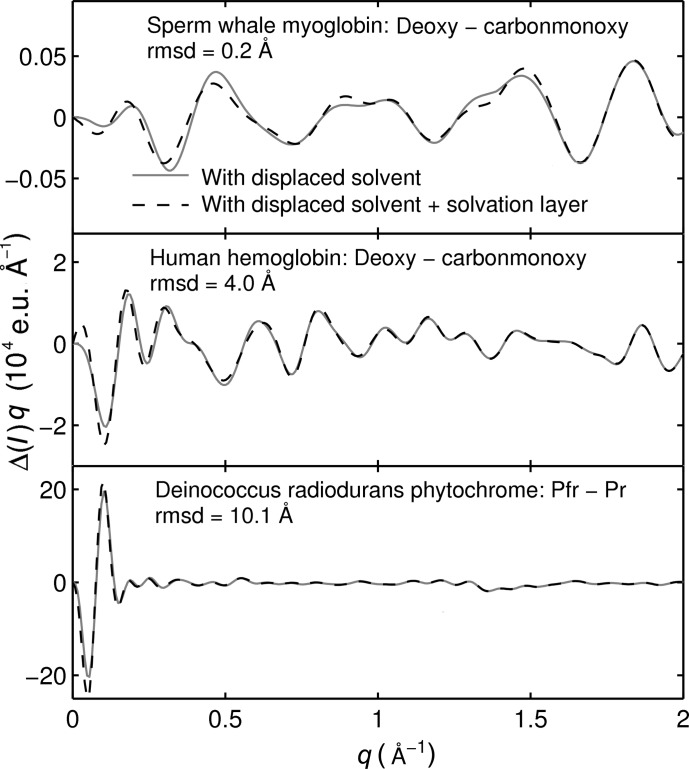
Solution difference scattering of three model systems with different magnitudes of conformational changes calculated with *CRYSOL* (Svergun *et al.*, 1995 ▶). The root-mean-square deviations (rmsd) for the respective conformers are shown in the panels. To evaluate the effect of displaced solvent and solvation layer scattering, two difference curves are shown: (i) atomic X-ray scattering with displaced solvent and (ii) atomic X-ray scattering with displaced solvent and solvation layer. The following structures were used (PDB codes in brackets): sperm whale deoxy myoglobin (2g0v, second state; Aranda *et al.*, 2006 ▶), sperm whale carbonmonoxy myoglobin (2g0r; Aranda *et al.*, 2006 ▶), human deoxy hemoglobin (2hhb) and human carbonmonoxy hemoglobin (1bbb); these structures were used without cofactors. The deinococcus radiodurans phytochrome solution structures were taken from Takala *et al.* (2014 ▶).

The calculations shown in Fig. 4 ▶ include the displaced-solvent term, but any excess electron density of the solvation layer was neglected. This is reasonable when considering difference scattering, as errors cancel to some degree when taking differences. The data in Fig. 5 ▶ demonstrate this. The difference scattering for three systems is shown: sperm whale myoglobin (deoxy and carbonmonoxy state), human hemoglobin (deoxy and carbonmonoxy state) and deinococcus radiodurans phytochrome (Pr and Pfr state). These systems were selected since they cover a wide range of magnitudes in conformational change as shown by the root-mean-square deviations of the respective structure pairs (*cf*. Fig. 5 ▶). The two solution difference scattering curves for each test system are computed by (i) considering the atomic scattering and the displaced solvent [as formulated in equation (4)], and (ii) additionally including the scattering due to the solvation layer. The data were calculated using *CRYSOL* (Svergun *et al.*, 1995 ▶), with its highest resolution (*L* = 50) and default parameters for the solvation shell and displaced solvent (Svergun *et al.*, 1995 ▶). The program approximates the solvation layer effect by assuming a uniform electron distribution around the protein that differs from bulk water by +10%, which is known to be inaccurate in the high-*q* region (Park *et al.*, 2009 ▶). However, the simple solvation layer treatment in *CRYSOL* can be used as a prototype to estimate the effect of a solvation layer model on the calculation of difference scattering. It is evident that the calculation with displaced solvent is in good agreement with the one that additionally models the solvation layer scattering for all three test systems.

### 3.3. Reliability of absolute X-ray scattering calculated from coarse-grained protein structures   

**Figure 6:**
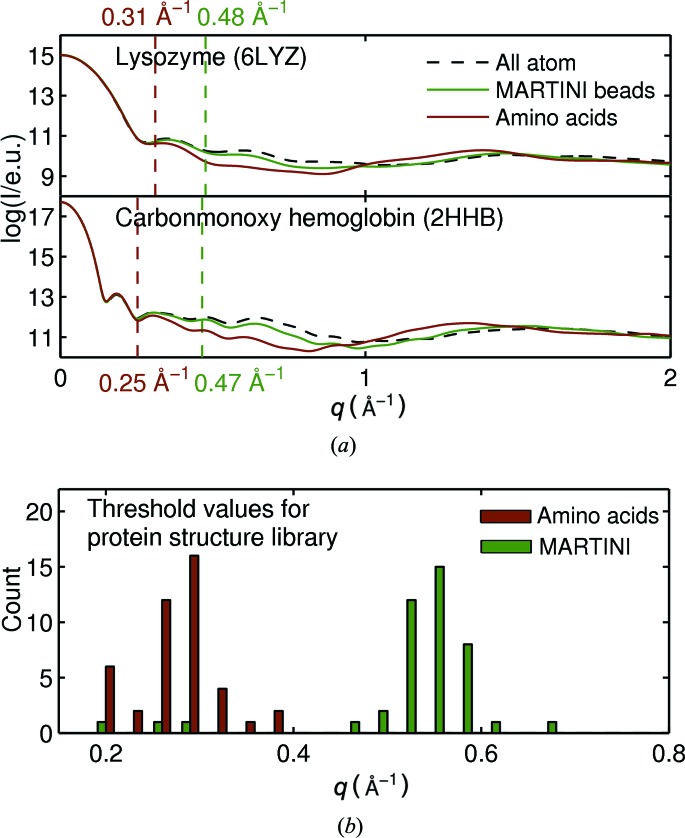
(*a*) Determination of *q*_threshold_ (see text for details) for reliable coarse-grained calculation of protein X-ray scattering for hen egg-white lysozyme (PDB code 6lyz) and carbonmonoxy hemoglobin (PDB code 2hhb). The cofactors were removed before evaluating the scattering. (*b*) For each structure of the protein structure library, the maximum *q* value with an error lower than 0.2% [according to equation (12)] was determined. The histogram illustrates the distribution of these *q*_threshold_ values for both coarse-grained methods (amino acid and MARTINI).

We now turn our attention to assessing the accuracy of the calculations of absolute X-ray scattering from coarse-grained structures. Fig. 6 ▶ shows the effect of coarse-graining on X-ray scattering calculations for a hen egg-white lysozyme [PDB code 6lyz (Diamond, 1974 ▶); Fig. 6 ▶(*a*)] and human carbon­mon­oxy hemoglobin [PDB code 2hhb, Fig. 6 ▶(*a*)]. These structures are not part of the protein structure library used to derive the coarse-grained form factors. For both structures, the agreement between the all-atom calculation and the coarse-grained calculations are good for low *q* and start to become worse at higher *q*. As expected, coarse-graining according to the MARTINI scheme agrees with the all-atom calculation to higher *q* than the amino acid approach. To quantify this agreement we use the average relative squared error in the range from 0 to *q*(*N*) with *N* being the number of data points in the respective range: 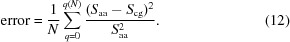
The dotted lines in Fig. 6 ▶ mark the maximum *q* value for the coarse-grained calculations (*q*_threshold_), for which the error [equation (12)] is smaller than 0.2%. The error limit of 0.2% is arbitrary but was chosen with respect to the absolute scattering curves of hen egg-white lysozyme (Fig. 6 ▶ *a*). For hen egg-white lysozyme and human carbonmonoxy hemoglobin, the *q*_threshold_ values are 0.31 and 0.25 Å^−1^, respectively, for the amino acid approach and 0.48 and 0.47 Å^−1^, respectively, for the MARTINI bead approach.

The *q*_threshold_ [equation (12)] values for the amino acid and the MARTINI approach for all proteins in the library are depicted as a histogram in Fig. 6 ▶(*b*). It is evident that the MARTINI coarse-grained calculations provide a wider *q* range (on average 0.53 Å^−1^) compared with the amino acid bead approach (on average 0.27 Å^−1^). This shows that the use of MARTINI beads significantly extends the range in which scattering can be reliably calculated compared with amino acid coarse-graining.

We note that the results presented in Fig. 6 ▶ do not include a model of the solvation layer around the protein. A number of sophisticated methods to incorporate this are already available, and this is critical for comparison with absolute experimental SAXS/WAXS data (Grishaev *et al.*, 2010 ▶; Park *et al.*, 2009 ▶; Bardhan *et al.*, 2009 ▶). However, the underlying physics, which is that a coarser representation of structure leads to a loss in resolution, is well captured in the model that was used to compute the data shown in Fig. 6 ▶.

## 4. Discussion   

The increasingly popular method of time-resolved WAXS requires advanced computational structural refinement schemes. In this paper, we have shown that coarse-graining the structures leads to a loss of accuracy at wide angles. Thus, when choosing the coarseness of the structural model, computational cost should be carefully balanced against the accuracy needed, a decision which critically depends on the *q* range of interest. For the case of human hemoglobin presented above, high-resolution crystal models deviate from experimental solution data for *q* > 0.4 Å^−1^. Thus a MARTINI representation and scattering model would be suitable for interpreting the available difference data up to *q* = 0.75 Å^−1^.

To the best of our knowledge, there are three refinement schemes for time-resolved X-ray scattering experiments of proteins. Ahn *et al.* (2009 ▶) have successfully applied a biased molecular dynamics simulation to time-resolved X-ray scattering data, Andersson *et al.* (2009 ▶) and Ahn *et al.* (2009 ▶) moved rigid bodies, and Kim, Lee *et al.* (2012 ▶) used *ab initio* determination of the three-dimensional structure. For these three approaches, a large number of X-ray scattering calculations had to be performed and this was the limiting factor in the studies. A treatment of larger proteins becomes prohibitively expensive. The MARTINI coarse-grained calculations offer a good compromise between accuracy in the X-ray scattering calculation and computational speed. The computation of X-ray scattering from coarse-grained protein structures on the MARTINI level is 50 times faster than the corresponding all-atom calculation (around 7–8 atoms go into the average MARTINI bead, 7^2^ = 49). This speed-up could break new ground for applying difference scattering-assisted structural refinement to larger proteins.^1^

When many trial structures are to be evaluated, using computationally demanding state-of-the-art methods to account for solvation effects is not feasible. We show here that less sophisticated solvation treatment can be used to reliably model difference WAXS. This is rationalized by the fact that the shape of the solvent shell does not change very much between different protein conformations, and that its contribution to the scattering simply cancels out in the difference scattering. Another advantage of using difference scattering as an experimental observable is that it is free of experimental artifacts stemming from incorrect subtraction of scattering from the buffer and the capillary. This means that the discrepancy between calculation and experiment significantly diminishes compared with standard SAXS/WAXS.

## 5. Conclusion   

The speed of computations of X-ray scattering from (bio)molecules can be controlled by coarse-graining the underlying structures. Our results provide the basis for matching the level of coarse-graining with the required resolution. When the beads contain entire amino acids and for the finer MARTINI scheme, we estimate reliability *q* ranges of 0–0.3 Å^−1^ and 0–0.5 Å^−1^, respectively. The MARTINI coarse-grained model thus covers the *q* range available in standard SAXS experiments and is 50 times faster than the all-atom calculation. When difference X-ray scattering is analyzed, for example, in time-resolved SAXS/WAXS, the reliability *q* range is significantly extended to 0.75 Å^−1^, which we showed for the model system human hemoglobin. We anticipate that the increased efficiency in computation of protein X-ray scattering will enable more comprehensive structural analyses in the growing field of time-resolved difference X-ray scattering of proteins. 

## Supplementary Material

Form factor library for MARTINI beads. DOI: 10.1107/S1600576714009959/aj5230sup1.pdf

Form factors for MARTINI beads. DOI: 10.1107/S1600576714009959/aj5230sup2.txt

## Figures and Tables

**Table 1:** Bead types used for the coarse-grained X-ray scattering calculations and their elemental formulae The sums of the atomic scattering factors at *q* = 0, corrected with the displaced-solvent model, are shown in the last columns. The form factors of all beads with *f*(*q* = 0) 0, marked in bold, were corrected as described in the text.

		Number of atoms					
AA	Bead	C	H	N	O	S	*f*(*q* = 0)
ALA	BB	3	5	1	1	0	9.04
ALA	BB	3	5	1	1	0	10.69
**ARG**	**SC1**	**3**	**6**	**0**	**0**	**0**	**2.79**
ARG	SC2	1	5	3	0	0	15.39
ASN	BB	2	2	1	1	0	10.69
ASN	SC1	2	4	1	1	0	9.25
ASP	BB	2	2	1	1	0	10.69
ASP	SC1	2	2	0	2	0	9.48
CYS	BB	2	2	1	1	0	10.69
CYS	SC1	1	2	0	0	1	8.44
GLN	BB	2	2	1	1	0	10.69
GLN	SC1	3	6	1	1	0	8.32
GLU	BB	2	2	1	1	0	10.69
GLU	SC1	3	4	0	2	0	8.55
GLY	BB	2	3	1	1	0	9.97
HIS	BB	2	2	1	1	0	10.69
**HIS**	**SC1**	**2**	**2**	**0**	**0**	**0**	**0.42**
HIS	SC2	1	1	1	0	0	5.95
HIS	SC3	1	1	1	0	0	5.95
ILE	BB	2	2	1	1	0	10.69
**ILE**	**SC1**	**4**	**9**	**0**	**0**	**0**	**4.44**
LEU	BB	2	2	1	1	0	10.69
**LEU**	**SC1**	**4**	**9**	**0**	**0**	**0**	**4.44**
LYS	BB	2	2	1	1	0	10.69
**LYS**	**SC1**	**3**	**6**	**0**	**0**	**0**	**2.79**
LYS	SC2	1	5	1	0	0	3.07
MET	BB	2	2	1	1	0	10.69
MET	SC1	3	7	0	0	1	5.86
PHE	BB	2	2	1	1	0	10.69
**PHE**	**SC1**	**3**	**3**	**0**	**0**	**0**	**0.63**
**PHE**	**SC2**	**2**	**2**	**0**	**0**	**0**	**0.42**
**PHE**	**SC3**	**2**	**2**	**0**	**0**	**0**	**0.42**
PRO	BB	2	1	1	1	0	11.41
**PRO**	**SC1**	**3**	**6**	**0**	**0**	**0**	**2.79**
SER	BB	2	2	1	1	0	10.69
SER	SC1	1	3	0	1	0	3.30
THR	BB	2	2	1	1	0	10.69
THR	SC1	2	5	0	1	0	2.37
TRP	BB	2	2	1	1	0	10.69
TRP	SC1	3	2	0	0	0	0.09
TRP	SC2	2	2	1	0	0	5.74
**TRP**	**SC3**	**2**	**2**	**0**	**0**	**0**	**0.42**
**TRP**	**SC4**	**2**	**2**	**0**	**0**	**0**	**0.42**
TYR	BB	2	2	1	1	0	10.69
**TYR**	**SC1**	**3**	**3**	**0**	**0**	**0**	**0.63**
**TYR**	**SC2**	**2**	**2**	**0**	**0**	**0**	**0.42**
TYR	SC3	2	2	0	1	0	4.53
VAL	BB	2	2	1	1	0	10.69
**VAL**	**SC1**	**3**	**7**	**0**	**0**	**0**	**3.51**

**Table 2:** PDB structures used for the determination of the average bead form factors

PDB entry	Atoms	Amino acids	MARTINI beads
1ARV	4845	336	682
1AVD	3854	247	555
1BP2	1842	123	276
1BPI	892	58	130
1COL	6006	394	804
1CSE	4827	337	685
1CSH	6765	435	952
1DHR	3527	236	493
1GAL	8700	581	1242
1HCB	3973	258	585
1HEL	1960	129	283
1HML	1946	123	277
1HRC	1672	104	236
1HRS	2775	174	385
1ISC	5852	384	862
1LPE	2364	144	310
1MOL	3124	188	440
1PNK	11708	750	1675
1PPN	3245	212	469
1RTP	4974	327	705
1SCS	3564	237	518
1SXC	4320	302	616
1THW	3031	207	445
1TOP	2466	162	338
1UBI	1231	76	163
1XYP	5636	378	856
1YMB	2411	153	343
2AAI	8212	529	1149
2CGA	7154	490	1022
2GST	7246	434	1036
2OHX	11278	748	1576
2PSG	5425	369	780
2SBL	25698	1614	3636
2ST1	3837	275	540
2TGA	3222	223	465
3EBX	920	62	139
3PGK	6376	415	878
3PTE	5163	347	728
4CMS	4854	320	704
4PEP	4672	325	679
6RAT	1857	124	273
7TIM	7556	494	1050

## Footnotes

1 The form factors are available from the IUCr electronic archives (Reference: AJ5230).
