# MARTINI bead form factors for the analysis of time-resolved X-ray scattering of proteins. Erratum

**DOI:** 10.1107/S1600576718007331

**Published:** 2018-05-18

**Authors:** Stephan Niebling, Alexander Björling, Sebastian Westenhoff

**Affiliations:** a Department of Chemistry and Molecular Biology, University of Gothenburg, Box 462, SE-40530 Gothenburg, Sweden

**Keywords:** X-ray solution scattering, proteins, coarse-graining, MARTINI, structural dynamics, small-angle X-ray scattering (SAXS), wide-angle X-ray scattering (WAXS), protein structure refinement

## Abstract

A typographical error in the article by Niebling, Björling & Westenhoff [*J. Appl. Cryst.* (2014), **47**, 1190–1198] is corrected.

There is a typographical error in Table 1 of the article by Niebling *et al.* (2014 ▸). The right entry for the second line should be

ARG BB 2 2 1 1 0 10.69

instead of

ALA BB 3 5 1 1 0 10.69.

The authors thank Darren Hsu for pointing out this error.
