# Supplementary material for: MARTINI bead form factors for the analysis of time-resolved X-ray scattering of proteins
Source: J Appl Crystallogr. 2014 Jun 14;47(Pt 4):1190–8. doi: 10.1107/S1600576714009959 (PMC4119947; doi:10.1107/S1600576714009959)
Supplement: Supplementary file 1 [file j-47-01190-sup1.pdf]

MARTINI bead form factors for the analysis of time-resolved X-ray scattering of proteins: Form factor library for MARTINI beads

Stephan Niebling, Alexander Björling and Sebastian Westenhoff[1]

[1]Department of Chemistry and Molecular Biology, University of Gothenburg, Box 462, SE-40530 Gothenburg, Sweden

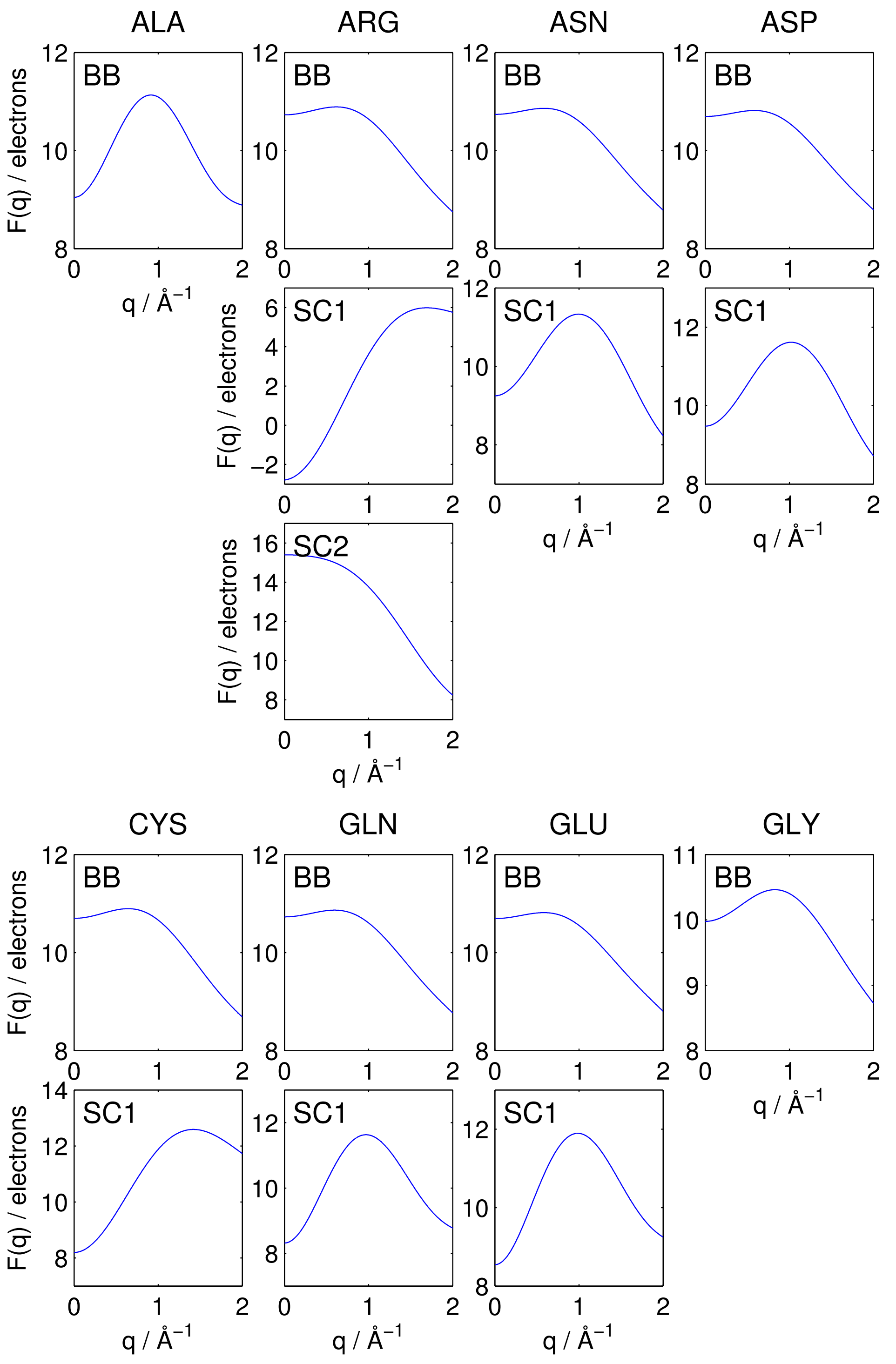
ALA
ARG
ASN
ASP
BB
SC1
SC2
CYS
GLN
GLU
GLY
F(q) / electrons
q / Å^{-1}

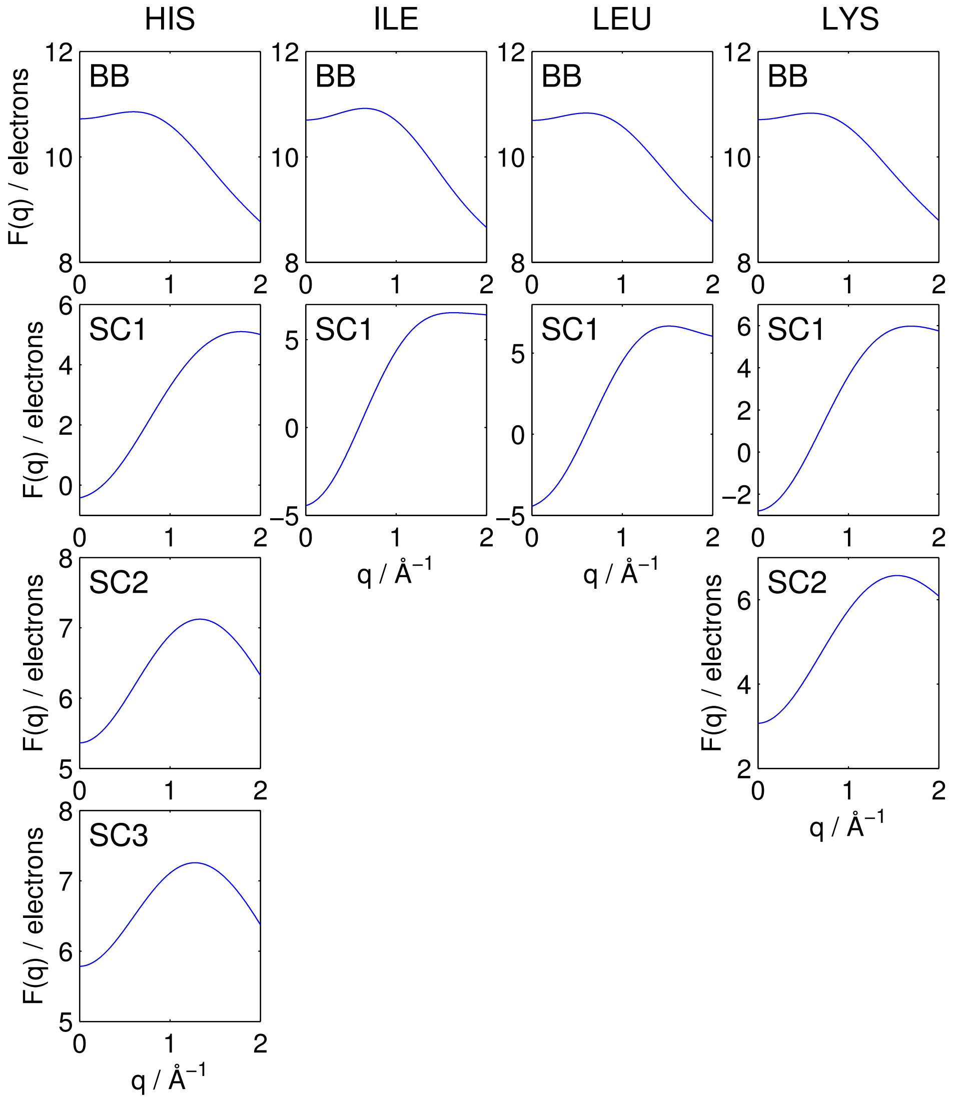
HIS
ILE
LEU
LYS
BB
SC1
SC2
SC3
F(q) / electrons
q / Å^{-1}

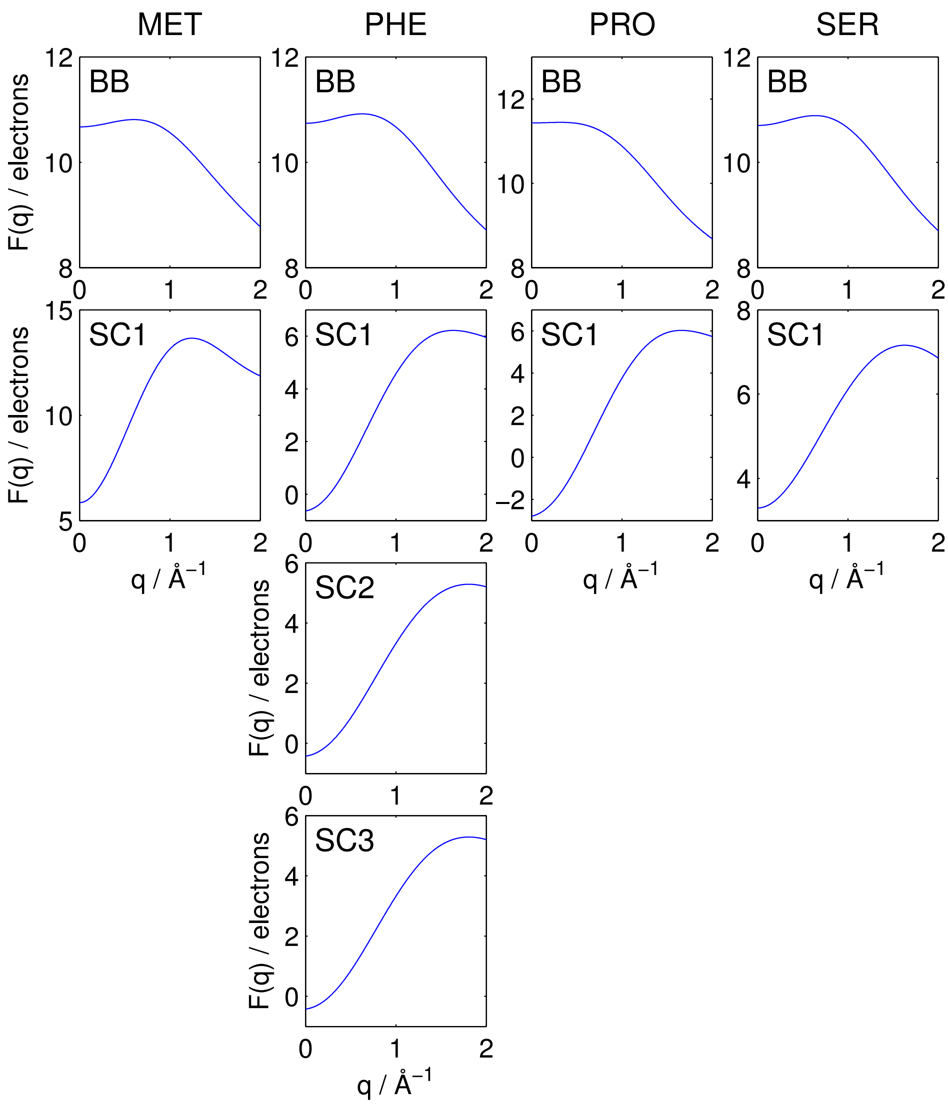
MET
PHE
PRO
SER
BB
SC1
SC2
SC3
F(q) / electrons
q / Å^{-1}

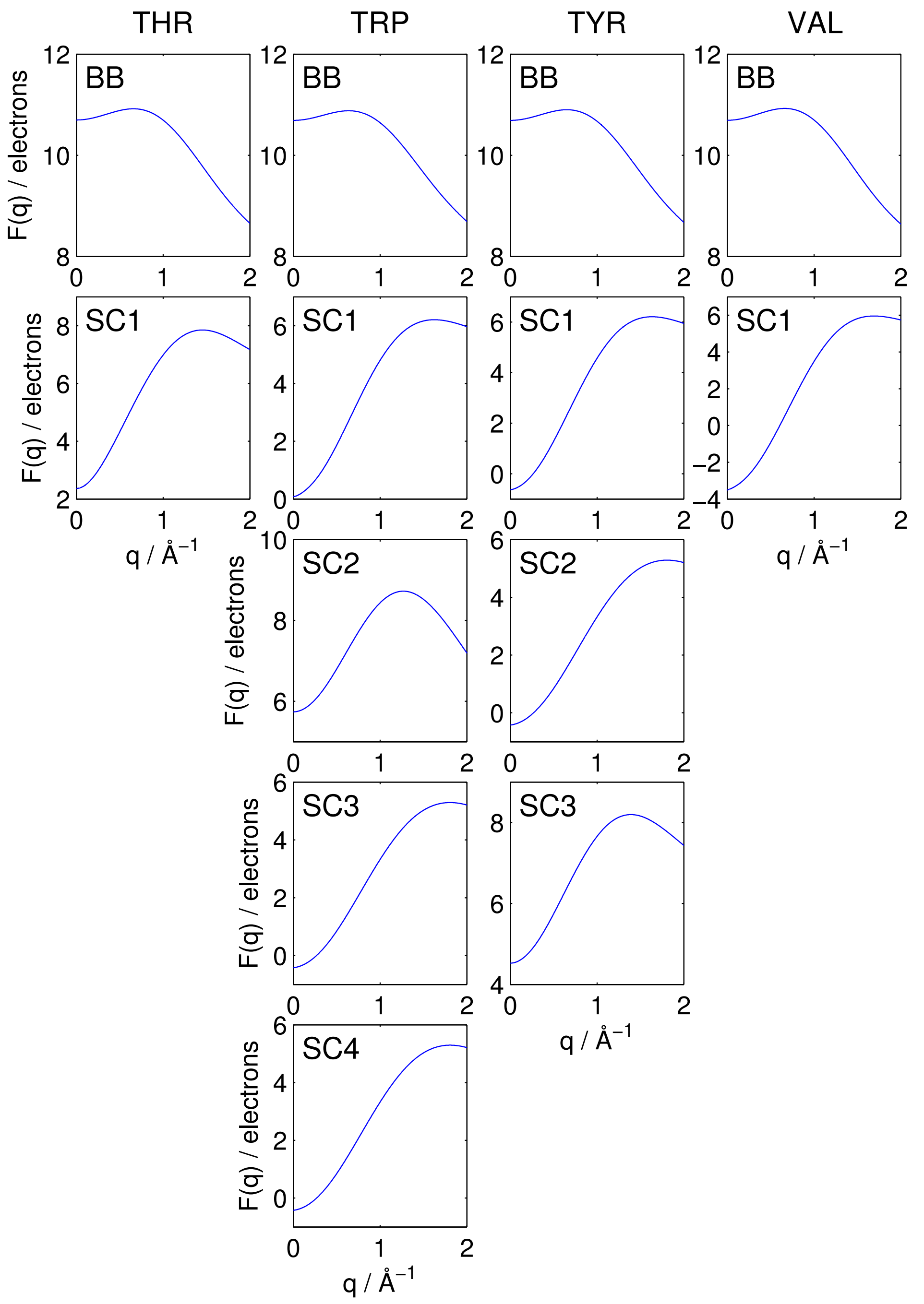
THR
TRP
TYR
VAL
BB
SC1
SC2
SC3
SC4
F(q) / electrons
q / Å^{-1}
